# HLA-Bw4 in association with KIR3DL1 favors natural killer cell-mediated protection against severe COVID-19

**DOI:** 10.1080/22221751.2023.2185467

**Published:** 2023-03-13

**Authors:** Ruihua Wang, Ying Sun, Bo-Hua Kuang, Xiao Yan, Jinju Lei, Yu-Xin Lin, Jinxiu Tian, Yating Li, Xiaoduo Xie, Tao Chen, Hui Zhang, Yi-Xin Zeng, Jincun Zhao, Lin Feng

**Affiliations:** a Department of Experimental Research, State Key Laboratory of Oncology in South China, Collaborative Innovation Center for Cancer Medicine, Guangdong Key Laboratory of Nasopharyngeal Carcinoma Diagnosis and Therapy, Sun Yat-sen University Cancer Center, Guangzhou, People’s Republic of China; b State Key Laboratory of Respiratory Disease at People’s Hospital of Yangjiang, Guangzhou Institute of Respiratory Health, the First Affiliated Hospital of Guangzhou Medical University, Guangzhou, People’s Republic of China; c Cancer Center, Union Hospital, Tongji Medical College, Huazhong University of Science and Technology, Wuhan, People’s Republic of China; d Cancer Center, Renmin Hospital of Wuhan University, Wuhan, People’s Republic of China; e Department of Biochemistry, School of Medicine, Sun Yat-sen University, Shenzhen, People’s Republic of China; f Institute of Human Virology, Key Laboratory of Tropical Disease Control of Ministry of Education, Guangdong Engineering Research Center for Antimicrobial Agent and Immunotechnology, Zhongshan School of Medicine, Sun Yat-sen University, Guangzhou, People’s Republic of China

**Keywords:** COVID-19, SARS-CoV-2, NK cells, Bw4 epitope, HLA-KIR interaction

## Abstract

Replicating SARS-CoV-2 has been shown to degrade HLA class I on target cells to evade the cytotoxic T-cell (CTL) response. HLA-I downregulation can be sensed by NK cells to unleash killer cell immunoglobulin-like receptor (KIR)-mediated self-inhibition by the cognate HLA-I ligands. Here, we investigated the impact of HLA and KIR genotypes and HLA-KIR combinations on COVID-19 outcome. We found that the peptide affinities of HLA alleles were not correlated with COVID-19 severity. The predicted poor binders for SARS-CoV-2 peptides belong to HLA-B subtypes that encode KIR ligands, including Bw4 and C1 (introduced by B*46:01), which have a small F pocket and cannot accommodate SARS-CoV-2 CTL epitopes. However, HLA-Bw4 weak binders were beneficial for COVID-19 outcome, and individuals lacking the HLA-Bw4 motif were at higher risk for serious illness from COVID-19. The presence of the HLA-Bw4 and KIR3DL1 combination had a 58.8% lower risk of developing severe COVID-19 (OR = 0.412, 95% CI = 0.187-0.904, *p *= 0.02). This suggests that HLA-Bw4 alleles that impair their ability to load SARS-CoV-2 peptides will become targets for NK-mediated destruction. Thus, we proposed that the synergistic responsiveness of CTLs and NK cells can efficiently control SARS-CoV-2 infection and replication, and NK-cell-mediated anti-SARS-CoV-2 immune responses being mostly involved in severe infection when the level of ORF8 is high enough to degrade HLA-I. The HLA-Bw4/KIR3DL1 genotype may be particularly important for East Asians undergoing COVID-19 who are enriched in HLA-Bw4-inhibitory KIR interactions and carry a high frequency of HLA-Bw4 alleles that bind poorly to coronavirus peptides.

## Introduction

Coronavirus disease 2019 (COVID-19) is caused by infection with a new coronavirus called severe acute respiratory syndrome coronavirus 2 (SARS-CoV-2). COVID-19 has a wide range of clinical presentations, from asymptomatic to severe pneumonia that can lead to respiratory failure and even death [1]. Many factors affect the clinical variability of COVID-19, especially older age and comorbidities [2]. In addition, genetic factors related to diversity in the immune response may also contribute to individual differences in susceptibility to COVID-19 [3–5].

The human leukocyte antigen (HLA) system is a complex of genes at the most highly polymorphic region in the human genome that have multiple important functions in immune regulation. HLA class I antigens are involved in presenting foreign antigens to CD8+ T cells, and some alleles serve as ligands for killer immunoglobulin-like receptors (KIRs) expressed on NK cells. As different allelic HLA molecules (allotypes) bind different peptides, HLA polymorphisms are supposed to potentially affect COVID-19 vulnerability, similar to many other infectious diseases. However, genome-wide association studies (GWAS) did not show a dominant effect of the HLA locus in general COVID-19 patients [6]; moreover, a study based on data from 6,919 infected people found that neither the HLA genotype nor viral epitope correlated with COVID-19 severity [7]. Additionally, the results of the association of HLA genotypes and COVID-19 incidence and severity in different populations and areas were divergent [7–13]. The discrepancy may be because HLA polymorphisms vary in different ethnic populations, and more importantly, the functional outcomes that result from HLA-I polymorphisms are complex, and the effects are conjoined by viral peptide avidity, surface expression, HLA receptors on T cells (TCRs) and NK cells (KIRs). Unlike SARS-CoV-1, SARS-CoV-2 has evolved a strategy to avoid the CTL response by degrading HLA-I through its unique ORF8 protein [14, 15], which may in part explain the lack of a strong association between HLA genotypes and COVID-19 outcome.

Killer cell immunoglobulin-like receptors (KIRs) are expressed on NK cell surface and act as sensors for HLA class I expression. KIRs have two or three immunoglobulin-like domains with either long (2DL, 3DL) or short (2DS, 3DS) tails. Long-tailed receptors are inhibitory, and short-tailed receptors are activating receptors. The interactions of inhibitory KIRs with HLA-I ligands have been well established. A subset of HLA-I acts as KIR ligands. HLA-C allotypes are ligands for inhibitory KIR2DL molecules and subdivided into C1 and C2, defined by amino acid variation at position 80 as Asp (C1) or Lys (C2). The C1 subsets are ligands for the inhibitory receptors KIR2DL2 and KIR2DL3, whereas the C2 subsets interact with the inhibitory receptor KIR2DL1 and the activating receptor KIR2DS1, but the binding of KIR2DS1 is substantially weaker than that of KIR2DL1. HLA-A and HLA-B allotypes with Bw4 motifs are ligands for KIR3DL1. HLA-A3 and HLA-A11 are ligands for KIR3DL2. The affinity of these interactions differs. The strongest HLA-KIR pairs include HLA-Bw4/KIR3DL1 and HLA-C2/KIR2DL1, followed by HLA-C1/KIR2DL2 and HLA-C1/KIR2DL3, and the HLA-A3, HLA-A11/KID3DL2 interaction is often peptide-dependent [16–18]. As each individual carries a unique set of HLA and KIR genes, significant variations in the NK cell repertoire exist between individuals and populations.

SARS-CoV-2 infection reduces HLA-I abundance at the cell surface through a unique SARS-CoV-2 gene encoding ORF8 [14]. According to the “licensing” model, NK cells acquire effector function by an education process mediated by inhibitory receptors that can bind to self-HLA class I molecules. NK cells expressing strong inhibitory receptors for self-HLA-I are functionally more responsive to stimulation, while NK cells lacking self-specific inhibitory receptors are hyporeactive [19, 20]. Education on the self allows NK cells to detect aberrant cells with low surface expression of HLA-I, which is common during viral infection. As HLA downregulation would stimulate the missing-self response of the NK cells and thereby launch lysis of the target cells, the NK cell-mediated antiviral response might be especially important for SARS-CoV-2 elimination when T cells lose recognition for target cells, and the differences in NK cell responsiveness and potency should underlie interindividual variations in COVID-19 susceptibility.

Here, we determined the influence of HLA class I genotypes and HLA-KIR combinations on the incidence and severity of COVID-19 in a Chinese population. Unexpectedly, some HLA-B molecules carrying the Bw4 or C1 motif that were specifically enhanced in frequency in East Asia had very poor binding affinities to SARS-CoV-2 CTL epitopes but did not increase the risk of disease severity. The reduced viral peptide binding affinity was compensated for by their role in ligating the NK cell inhibitory receptors. Our data illustrated a protective role of HLA-inhibitory KIR interactions in the immune response against SARS-CoV-2 when HLA-I is degraded by ORF8. This finding was consistent with recent studies indicating that missing strong inhibitory signals during NK cell development resulted in poor activation potential in the event of viral infection that is able to downregulate HLA-I, such as SARS-CoV-2.

## Materials and methods

### Patient enrolment

This study included 105 laboratory-confirmed COVID-19 cases and 413 healthy controls from a Chinese ethnicity population-based random sample. Healthy controls were recruited from October 31, 2019, to August 6, 2020, and matched for sex and geographic area with COVID-19 patients. The laboratory-confirmed COVID-19 patients (aged ≥22 years) and healthy controls were mainly from South China, including Guangdong, Hubei, and Guizhou provinces. The patients were hospitalized between January 26 and May 4, 2020. The detailed information of patients and controls including gender, age, district, underlying diseases, and their HLA-I and KIR genotypes are provided in **Tables S1**. The main demographic characteristics of the controls and cases are listed in **Tables S2** and **S3**. Patients with severe pneumonia who were admitted to the ICU and required mechanical ventilation were enrolled in the severe illness group. Patients with a mild clinical presentation (primarily fever, cough, malaise, and headache, including no pneumonia or mild pneumonia) were enrolled in the mild illness group. Among 105, 57 patients were classified as having mild cases (including asymptomatic, mild, and moderate COVID-19), and 48 patients were classified as having severe cases (including critical and severe COVID-19). The present study had IRB approval from the Health Commission of Guangdong Province (2020-85). Written informed consent was obtained from all participants.

### HLA and KIR genotyping

Genomic DNA was extracted from blood samples using the QIAamp DNA Blood Mini Kit (Qiagen, Germany) according to the manufacturer’s protocol. The HLA Laboratory, CapitalBio Corporation (Beijing, China) performed HLA genotyping at a 4-digit resolution level for the HLA-A, HLA-B, and HLA-C loci using the Sanger sequence-based typing method (PCR-SBT). At least exons 2, 3, and 4 were amplified with locus-specific PCR primers for HLA-A, HLA-B, and HLA-C, respectively, and the SBT protocol was described in detail earlier [21]. KIR genotyping was performed using PCR with sequence-specific primers as described previously [22]. HLA typing was combined with KIR typing to stratify patients according to predicted KIR-ligand interactions.

### Peptide-HLA class I binding affinity predictions

Protein sequences from SARS-CoV-2 proteomes were obtained from the NCBI database under accession number NC_045512.2. The HLA class I sequences were extracted from the IPD-IMGT/HLA database (version 3.41.0). HLA binding affinity predictions were performed in the Immune Epitope Database (IEDB) with peptide lengths of 8∼14 aa [23]. HLA alleles that were unable to bind any SARS-CoV-2 S/M/N/E peptides with high affinity (IC50 ≤ 100 nM) were defined as weak binders (WBs), otherwise were defined as strong binders (SBs).

### Statistical analysis

The distributions of KIR genes, HLA ligands, and KIR-HLA combinations between the study groups were estimated by Pearson’s chi-square test, continuity correction (when the number of subjects in a cell was <5), or Fisher’s exact test (when the number of subjects in a cell was <1). The Bonferroni method was used for multiple comparison correction. For alleles associated with COVID-19 severity, the odds ratio (OR) was calculated with a 95% confidence interval (CI) using logistic regression, adjusted for sex and age, hypertension, coronary artery disease, and diabetes. A *p* value < 0.05 was defined as statistically significant.

### NK cell degranulation assay

Human PBMCs were isolated from the blood of healthy donor using Ficoll® Paque Plus (GE Healthcare, Cat #17-1440-03) density centrifugation. PBMCs (2 × 10^6^/mL) were stimulated overnight with 100 U/mL recombinant human IL-2 (PeproTech, Cat #200–02). Akata cells 48 h after transfection with ORF8 or EBV genes (BNLF2a or BILF1) were used as target cells. The positive control was co-incubated with K562 cells or treated with phorbol-12-myristate-13-acetate (PMA) and ionomycin (Leukocyte Activation Cocktail, with BD GolgiPlug™, BD Biosciences, Cat #500583).1 × 10^5^ target cells and 2 × 10^6^ PBMCs were resuspended at an effector-to-target (E:T) ratio of 20:1 in 200 μL fresh RPMI 1640 complete medium supplemented with 5 μL APC anti-human CD107a antibody (H4A3, Biolegend, Cat #328620). Cells were incubated in 96 well U-plate for 1 h at 37 °C and 5% CO2, 5 μg/mL brefeldin A solution (Biolegend, Cat #420601) was added and the cells were incubated at 37 °C and 5% CO2 for another 4 h. Cells were washed twice in PBS containing 0.5% (wt/vol) BSA and then stained for 30 min on ice with Brilliant Violet 650™ anti-human CD19 (BioLegend, Cat #363026) and PE/Cyanine7 anti-human CD56 (BioLegend, Cat #318318) for flow cytometry analysis.

### Flow cytometry

For analysis of the surface expression of HLA-I and HLA-II, cells were stained for 30 min in PBS containing 0.5% (wt/vol) BSA with the indicated antibodies on ice. The following antibodies were used: anti-HLA-A, HLA-B, and HLA-C (W6/32, Biolegend, Cat #311409), anti-HLA-DR, DP, and DQ (Tü39, Biolegend, Cat #361716), anti-HLA-A2 (BB7.2, Biolegend, Cat #343308), anti-HLA-B Bw4 (REA274, Miltenyi, Cat #130-103-848), and anti-HLA-C (DT-9, Biolegend, Cat #373307). Flow cytometry data were acquired on a Beckman CytoFLEX-S flow cytometer, and the data were analyzed using FlowJo software.

## Results

### HLA-I polymorphisms in SARS-CoV-2 susceptibility

A total of 105 COVID-19 cases from South China hospitalized between January 26 and May 4, 2020, and 413 healthy controls matched for geographic distribution and sex were enrolled in this study. The median age at diagnosis of severe COVID-19 was 56 years, and that of mild COVID-19 was 45 years. The detailed information of patients and controls were listed in **Table S1**. The male to female ratios were 2:1 in severe cases and nearly 1:1 in mild cases (**Table S2**). Consistent with previous reports, elderly patients with comorbidities were more vulnerable to severe COVID-19 (**Table S3,** median age of 56 in severe cases vs. 45 in mild cases, *p *= 3.450E-4).

**Figure 1.**
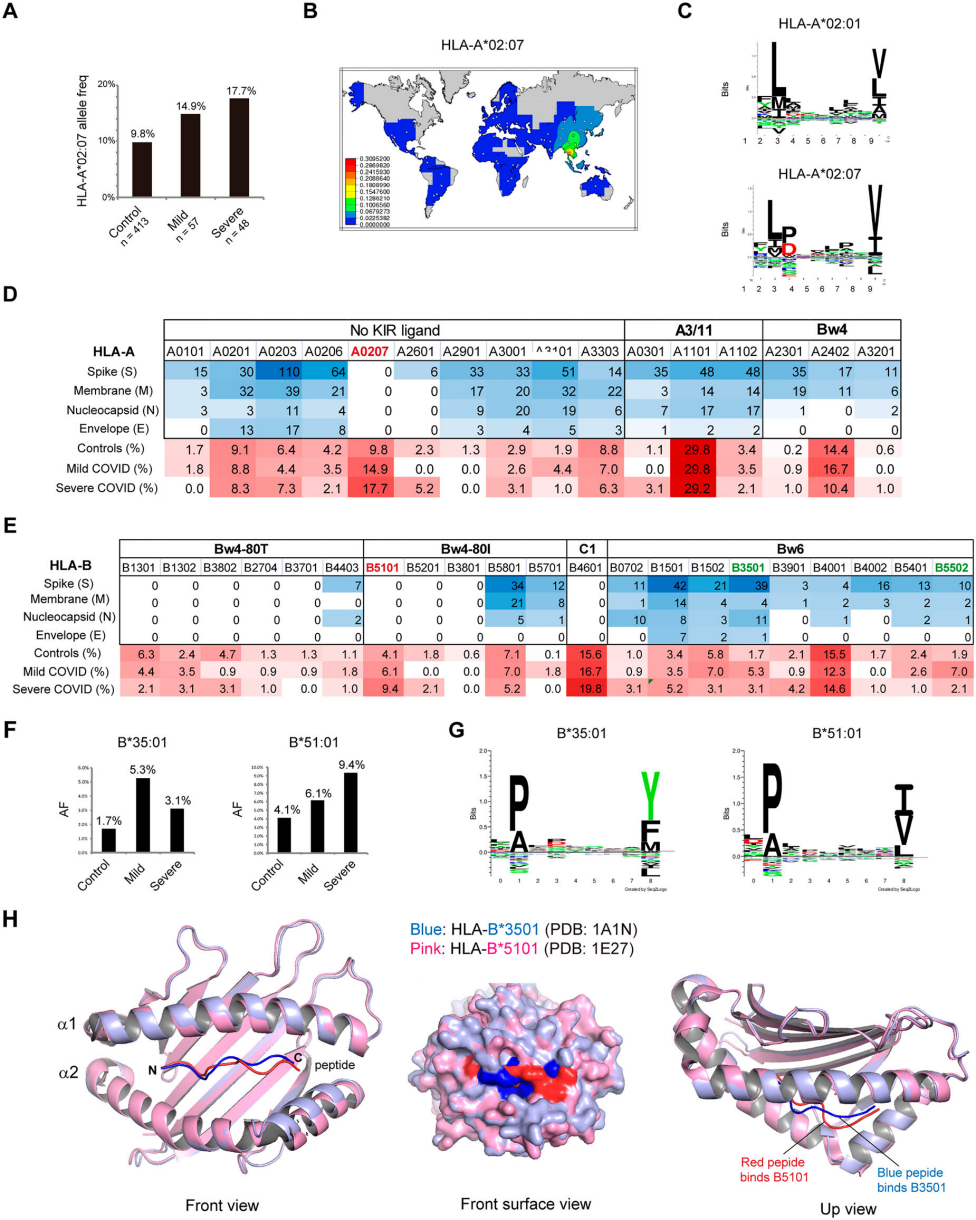
**COVID-19 risk-associated HLA class I alleles in South China**. **A**) Increased frequency of HLA-A*02:07 in COVID-19 patients compared to controls. Details of each group are listed in Tables S1-3. **B**) Distribution of the HLA-A*0207 allele worldwide. Image from http://pypop.org/popdata. **C**) Sequence logo showing the amino acid preference for peptide binding to HLA-A*02:01 and A*02:07 constructed with NetMHC 4.0. A*0207 has an additional restriction on peptides with D/P at P3 resulting from the Y99C mutation in the D pocket. **D**-**E**) Predicted HLA binding of peptides with strong affinity (IC50 ≤ 100 nM) from a set of all possible 9∼14-mers from the SARS-CoV-2 proteome according to IEDB prediction. The allele frequencies (AF) of the HLA-A allele in Chinese healthy controls and COVID-19 patients are shown at the bottom. **F**) Allele frequencies of HLA-B alleles that were distributed significantly differently in healthy controls and COVID-19 patients with mild or severe illness. **G**) Sequence logo showing the amino acid preferences for peptide binding. The binding motifs were from NetMHCpan-4.0. **H**) Overlay of the crystal structure of the protective allele HLA-B*35:01 (blue, PDB ID: 1A1N) and the risk allele HLA-B*51:01 (pink, PDB ID: 1E27) complexed with their peptides. Structures were visualized using the PyMOL Molecular Graphics System. The peptide-binding cleft was composed of the α1 and α2 regions.

To investigate the role of HLA-I molecules in susceptibility to COVID-19, we genotyped HLA class I (A, B and C) alleles by PCR-based, high-resolution genotyping and compared the HLA allotypes between COVID-19 patients and healthy controls. The allelic frequency distribution of HLA was largely similar between the case and control groups. Among HLA-I allotypes, HLA-A*02:07 was the most significantly increased allele in COVID-19 patients compared with controls (16.2% vs. 9.8%, OR = 1.796, 95% CI = 1.061-3.040, *p *= 0.009). Moreover, the frequency of A*02:07 was highest in severe COVID-19 patients, followed by mild patients (Figure 1A).

HLA-A*02:07 is the most common HLA-A2 subtype among southern Chinese individuals but is absent in most parts of the world (Figure 1B). The A*02:07 allele differs from the A*02:01 allele by a single amino acid residue (Y99C) that influences the affinity of peptide binding pockets in the HLA molecule (Figure 1C). We performed *in silico* prediction of HLA class I-viral peptide binding affinity using the Immune Epitope Database IEDB (www.iedb.org) for SARS-CoV-2 proteome of the structural proteins spike (S), nucleocapsid (N), membrane (M) and envelope (E), because structural proteins are the dominant targets of T-cell responses [24]. Among HLA-A alleles common in Asia, A*02:07 did not bind any SARS-CoV-2 epitopes with strong affinity, in sharp contrast to other A*02 allotypes (Figure 1D). However, the differences in A*02:07 allele frequency between patients and controls did not reach statistical significance after Bonferroni’s correction (*Pc* = 0.244).

HLA-A and HLA-B alleles are major HLA class I molecules engaged in CD8+ T-cell activation. We compared HLA-B allele frequencies in COVID-19 patients and controls (Figure 1E). Among HLA-B alleles, HLA-B*35:01 (5.3% vs. 1.7%, OR = 3.428, 95% CI  = 1.213-9.690, *p* = 0.033) and B*55:02 (7.0% vs. 2.9%, OR = 2.506, 95% CI = 1.036-6.066, *p* = 0.046) were overrepresented in mild COVID-19 patients compared with controls, whereas HLA-B*51:01 was more prevalent in severe cases than controls (9.4% vs. 4.1%, OR = 2.631, 95% CI  = 0.901-7.685, *p* = 0.040). Two out of three alleles have been published previously. HLA-B*35:01 was reported to be strongly associated with reduced disease duration in mild and moderate COVID-19 in a German population [25], and the risk effect of HLA-B*51:01 has also been discovered by a GWAS in Chinese COVID-19 [9]. While the prevalence of the three alleles fell below statistical significance after Bonferroni correction, the B*51:01 allele tended to increase COVID-19 incidence as A*02:07, and the B*35:01 and B*55:02 alleles seemed to be protective against COVID-19 severity (Figure 1F and **Table S4**).

We wondered whether different peptide binding preferences account for the association of certain HLA-B alleles with COVID-19 susceptibility. Surprisingly, both the protective allele HLA-B*35:01 and the risk allele HLA-B*51:01 belong to the HLA-B5 cross-reactive group [26]. Both allotypes had preferences for proline and alanine at position 2 (P2), but the preferences at the peptide C-terminal (PC) residues differed. B*51:01 recognizes peptides carrying aliphatic hydrophobic residues I/V/L at PC, while B*35:01 had a preference for aromatic residues Y/F at PC (Figure 1G). Crystallography studies showed that despite very similar binding conformations at the N-termini of the peptides when bound to their cognate HLAs, the two HLA alleles adopted substantially different conformations at the C-termini of the peptides. Compared to the risk allele B*51:01 (red), the protective B*35:01 (blue) possessed a deeper pocket within the binding cleft as pocket F, which allowed the peptide residue side chain to bury deeply within the F-pocket (Figure 1G). In contrast, B*51:01 had a shadow F-pocket, which may account for the risk effect of this allele and the low affinity for the SARS-CoV-2 proteome (Figure 1E) (see below).

### Preference of aromatic and branched-chain amino acids at the C-termini of SARS-CoV-2 CD8+ T epitopes and implications for HLA-I polymorphisms in COVID-19 susceptibility

**Figure 2.**
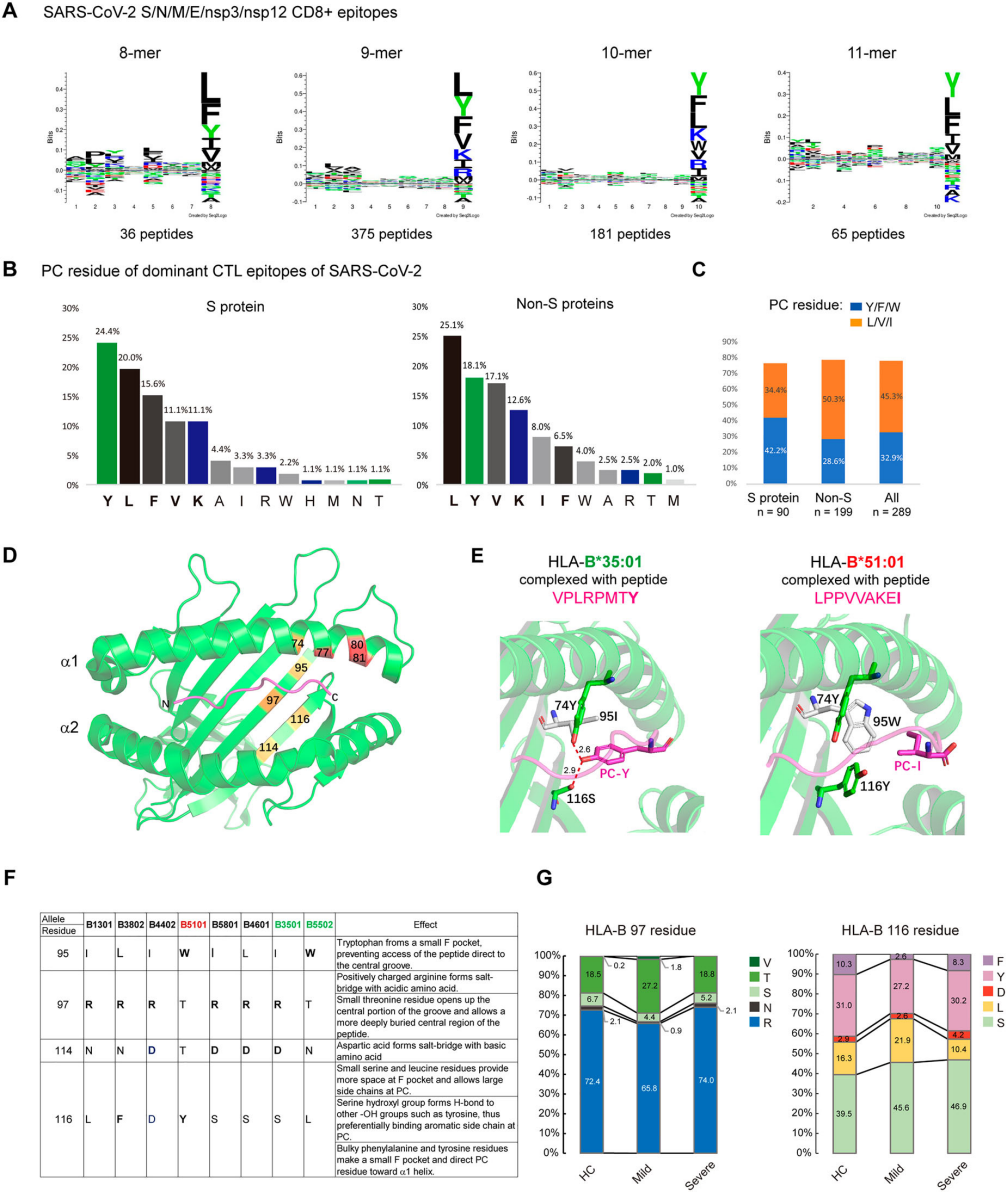
**Preference of aromatic and large aliphatic residues at the C-termini of SARS-CoV-2 CD8 + epitopes. A**) Sequence logo plot of the residues in the 8-, 9-, 10-, and 11-mer blocks of SARS-CoV-2 CD8 epitopes generated using Seq2Logo. Bits represent the relative frequencies of amino acids. **B**) Amino acids at the C-terminal residues of immunodominant CD8 epitopes of the spike protein and other proteins of SARS-CoV-2. C) Percentages of imunodominant CD8 epitopes that end with F/Y/W or L/V/I residues. **D**) Locations of F-pocket key residues in the HLA-I protein, corresponding to residues 74, 77, 80, 81, 95, 97, 114 and 116. The F-pocket residues also located in the Bw4 motif that interact with KIR are shown in red. **E**) Structures of the protective allele B3501 and the risk allele B5101 bound with their peptides. The structures are the same as those in Figure 1H. **F**) Amino acid residues at the key positions within the F-pocket of some HLA-B alleles and the effects of polymorphisms on peptide binding. **G**) Polymorphic amino acid residue composition of HLA-B at positions 97 and 116 in the three groups.

Overall, no strong allelic effects have been observed in COVID-19. To gain insight into the connection between HLA binding clefts and SARS-CoV-2 epitope motifs, we analyzed the published SARS-CoV-2 human CD8 T-cell epitope database [24]. Structural proteins are broadly recognized by T cells, and other proteins, such as nsp3 and nsp12, are also broadly recognized. In total, 657 epitopes of S/N/M/E and nsp3/nsp12 proteins were analyzed, with peptide lengths spanning from 8- to 11-mers. Intriguingly, alignment analysis revealed that only the C-terminal residues of the epitopes had a consensus sequence, with a strong preference for the aromatic amino acids tryptophan (Y) and phenylalanine (F) and branched-chain amino acids, including leucine (L), valine (V) and isoleucine (I), and to a lesser extent, the basic amino acids lysine (K) and arginine (R), which were depicted as sequence logos (Figure 2A). The PC specificities were also observed in the immunodominant CTL epitopes [24]. Similarly, aromatic amino acids Y/F and branched aliphatic amino acids L/V/I were overrepresented at the C-termini of the immunodominant epitopes (Figure 2B). Of note, epitopes of the Spike protein had a strong preference for Y/F at PC, and epitopes of nonspike proteins preferred branched-chain amino acids L/V/I at PC (Figure 2B and C).

HLA-I possesses six binding pockets within the binding cleft named pockets A-F, which allow residue side chains to anchor each pocket. The peptide C-terminal (PC) residue is engaged by the F-pocket of HLA class I. The F-pocket consists of residues 74, 77, 80, 81, 84, 95, 97, 114, 116, 123, 133, 143, 146, and 147, and the locations of some residues are marked in Figure 2D. We compared the F-pockets of the protective allele B*35:01 and the risk allele B*51:01. The protective allele B*35:01 possesses a polar residue serine (S) at position 116, which mediates hydrogen bond formation with the PC Tyr-OH (Figure 2E, left). In contrast, the risk allele B*51:01 carries a bulky tyrosine (Y) residue at position 116. Although Tyr is also a polar amino acid and carries a hydroxyl (-OH) group, similar to Ser, the side chain of Tyr is much larger than that of Ser. In addition, another aromatic residue, tryptophan (W), is located at position 95 of B*51:01, together with 116Y, making a much smaller F-pocket and directing the PC residue toward the α1 helix rather than allowing access to the bottom of the central groove and H-bond formation (Figure 2E, right and **2F**).

We then compared the amino acid residues within the F-pocket of HLA-B in controls and patients. Unexpectedly, F-pocket polymorphisms were similar between controls and severe COVID-19 patients but displayed a different pattern in mild patients. Mild patients had significantly fewer aromatic amino acids (F/Y) but more small amino acids (L/S) at position 116 than controls as well as severe group (F/Y: control 41.3%, mild 29.8%, severe 38.5%; L/S: control 55.8%, mild 67.6%, severe 57.3%). In addition, large residues (R/N) at position 97 were also decreased in the mild group compared with the other two groups, albeit to a lesser extent (R/N: control 74.5%, mild 66.7%, severe 76.1%; V/T/S: control 25.4%, mild 33.4%, severe 24.0%) (Figure 2G). Arginine (R) and asparagine (N) at position 97 make the central region solvent accessible, while small residues (T/S/V) open up the central portion of the groove and allow a more deeply buried central region of the peptide. Thus, the structural features of HLA class I molecules that are able to bind the SARS-CoV-2 CD8 epitope tightly appear to involve the F pocket of the antigen-binding cleft, and the alleles with larger F-pockets seemed to be protective against COVID-19. However, such superior peptide binding did not confer protection against severe COVID-19, indicating that in addition to HLA-I-medicated CTLs, other antiviral immune responses may be involved in combating SARS-CoV-2 infection.

### A subset of HLA-B alleles encoding the Bw4 epitope and HLA-B*46:01 carrying the C1 epitope bind poorly to SARS-CoV-2 peptides but do not contribute to COVID-19 severity

We noticed that in addition to B*51:01, a number of HLA-B alleles were also predicted to be poor binders for the SARS-CoV-2 proteome. Strikingly, all weak binders encoding a KIR-interacting motif, Bw4 or C1, and the risk allele B*51:01 belonged to the Bw4-80I subtype (Figure 1E). The Bw4 epitope acts as a ligand for NK cells through KIR3DL1 receptor interaction, and is further classified with Bw4-80I and Bw4-80 T according to the amino acid at position 80. Of HLA-B alleles carrying the Bw4-80 T motif (B*13, B*27, B*3701 and B*44), none of them except B*44:03 were predicted to be able to bind SARS-CoV-2 structural protein peptides with IC50 ≤ 100 nM. Among HLA-B-Bw4-80I alleles, B*38:01, B*51:01 and B*52:01 were unable to bind SARS-CoV-2 S/M/N/E peptides with high affinities, while B*58:01 presented a considerable number of SARS-CoV-2 peptides. In contrast, all of the Bw6 motif-containing HLA-B alleles common in China were able to present SARS-CoV-2 peptides to different extents (Figure 1E).

**Figure 3.**
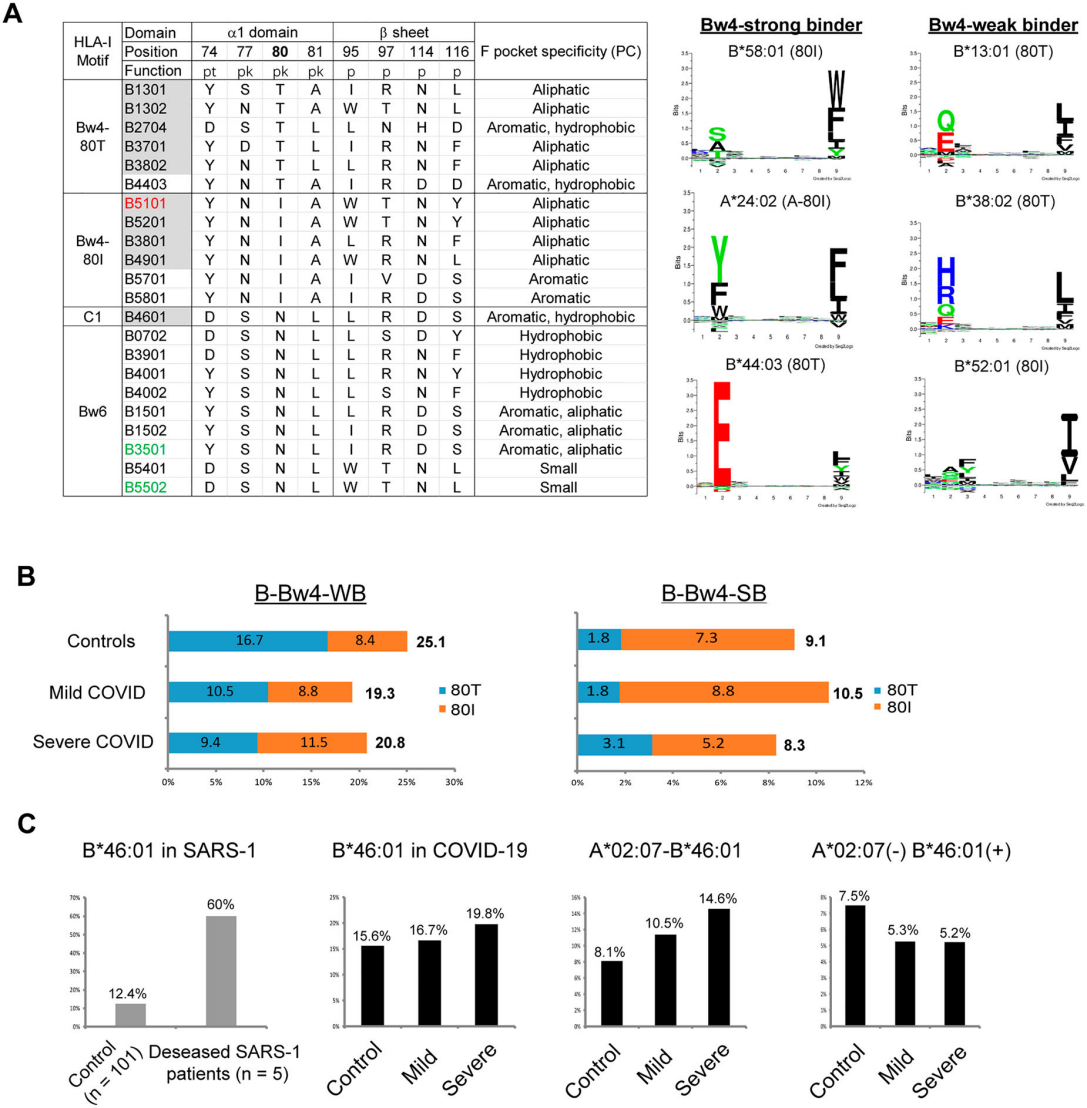
**HLA-B alleles with Bw4 or C1 motifs bind poorly to COVID-19 peptides and do not contribute to COVID-19 severity. A**) (Left) Key residues of the F-pocket and the amino acid specificities of the F- and B-pockets of HLA-B alleles classified by the KIR-binding motifs (Bw4-80 T, Bw4-80I and C1). HLA alleles that were predicted to lose SARS-CoV-2 peptide binding capacities were in gray background and were defined as weak binders, and alleles able to bind strongly to SARS-CoV-2 antigens were defined as strong binders. Residues that make contact with peptide (p), T-cell receptor (t) and killer cell immunoglobulin-like receptor (k) are indicated. (Right) Sequence logo showing the amino acid preferences for peptide binding of strong and weak binders of HLA-I with the Bw4 motif. Anchor motif of representative HLA-Bw4 alleles with predicted strong or weak binding capacities to SARS-CoV-2 peptides. Acidic (red), basic (blue), hydrophobic (black), and polar (green). The main differences between strong and weak binders lie in the P9 selectivity conferred by the F-pocket of HLA-I. The binding motifs were from NetMHCpan-4.0. **B**) Percentages of strong and weak binders of HLA-I with the Bw4 motif in healthy controls and COVID-19 patients with mild or severe symptoms. **C**) The increased HLA-B*46:01, which carries the C1 motif, was caused by linkage disequilibrium (LD) with HLA-A*02:07 in COVID-19. The HLA-B*46:01 frequency data for the deceased SARS-CoV-1 patients and controls (gray) in Taiwan are from [38].

We analyzed the binding specificity of pockets B and F, as the two pockets are strong determinants of peptide binding. We found that the P2 anchor residues varied within strong and weak binders, which is in line with the observation that there was a lack of a P2 consensus motif in SARS-CoV-2 epitopes (Figure 2A). In contrast, anchor residues at PC had clear differences in peptides bound to strong or weak binders of HLA-Bw4. Strong binders (SBs) seem to have a broader repertoire at PC, which could accommodate both aromatic and aliphatic amino acids, but weak binders (WBs) are only able to anchor some aliphatic residues, such as L/V/I, at PC, e.g. a subset of amino acids anchored by SBs (Figure 3A**, left**).

Considering the overrepresentation of tyrosine and phenylalanine (Y/F) at the PC of SARS-CoV-2 CTL epitopes (Figure 2A and B), we suggest that the differences in accommodation with aromatic amino acids at the carboxy-terminal position of the peptide may be a key distinction between SBs and WBs for the SARS-CoV-2 proteome. Comparing key residues in the F-pocket, weak binders (shadowed in gray) possess more bulky amino acids, such as 116F/Y, 95W and 114N, and strong binders have more polar and small amino acids at the same position, such as 116D/S, 95I/L and 114D (Figure 3A**, right**). As a result, Bw4-WBs were unable to accommodate aromatic and polar residues on PC.

Individuals with the HLA genotype encoding poor binders for SARS-CoV-2 epitopes are supposed to be detrimental because of insufficient T-cell activation, and such poor binders should be overrepresented in patients with severe illness. However, the frequencies of HLA-B-Bw4 alleles, either strong or weak binders for SARS-CoV-2 peptides, were both decreased in severe COVID-19. In contrast, the frequencies of SBs were increased and WBs were decreased in COVID-19 mild patients, in accordance with F-pocket characteristic changes that were found only in mild patients (Figure 2F).

In addition to a subset of HLA-Bw4 allotypes binding poorly to SARS-CoV-2 peptides, B*46:01, an unusual HLA-B allotype allele carrying the C1 motif, was also a poor binder for SARS-CoV-2 peptides [27]. The B46 allele is a product of interlocus recombination between HLA-B*15:01 and C*01:02. This hybrid allele acquired new properties in peptide selection at P1, P2 and P8 [28], which could not accommodate any SARS-CoV-2 peptides by prediction. B*46:01 had been strongly associated with the severity of the 2003 SARS pandemic [29] but was only slightly increased in COVID-19 patients (HC, mild and severe cases: 15.6%, 16.7% and 19.8%) (**Table S6**), in sharp contrast to observations in deceased SARS-1 patients (12.4% vs. 60%, data from [29]) (Figure 3C, right). Moreover, the risk effect of B*46:01 in COVID-19 was largely caused by its strong linkage disequilibrium (LD) with A*02:07, as in the absence of HLA-A*02:07, the frequency of HLA-B*46:01 alone was reduced in COVID-19 patients (Figure 3C. left).

The lower incidence of poor binders for SARS-CoV-2 peptides in COVID-19 severe patients, including HLA-Bw4 weak binders and HLA-B*46:01 alone, raises the possibility that the reduced SARS-CoV-2 antigen-presenting capacity by some HLA-I allotypes encoding KIR binding motifs might be compensated by their role in NK cell licensing.

### Reduced HLA-Bw4/KIR3DL1 combination in patients with severe COVID-19 and increased HLA-C2/KIR2DL1 pair in mild COVID-19

**Figure 4.**
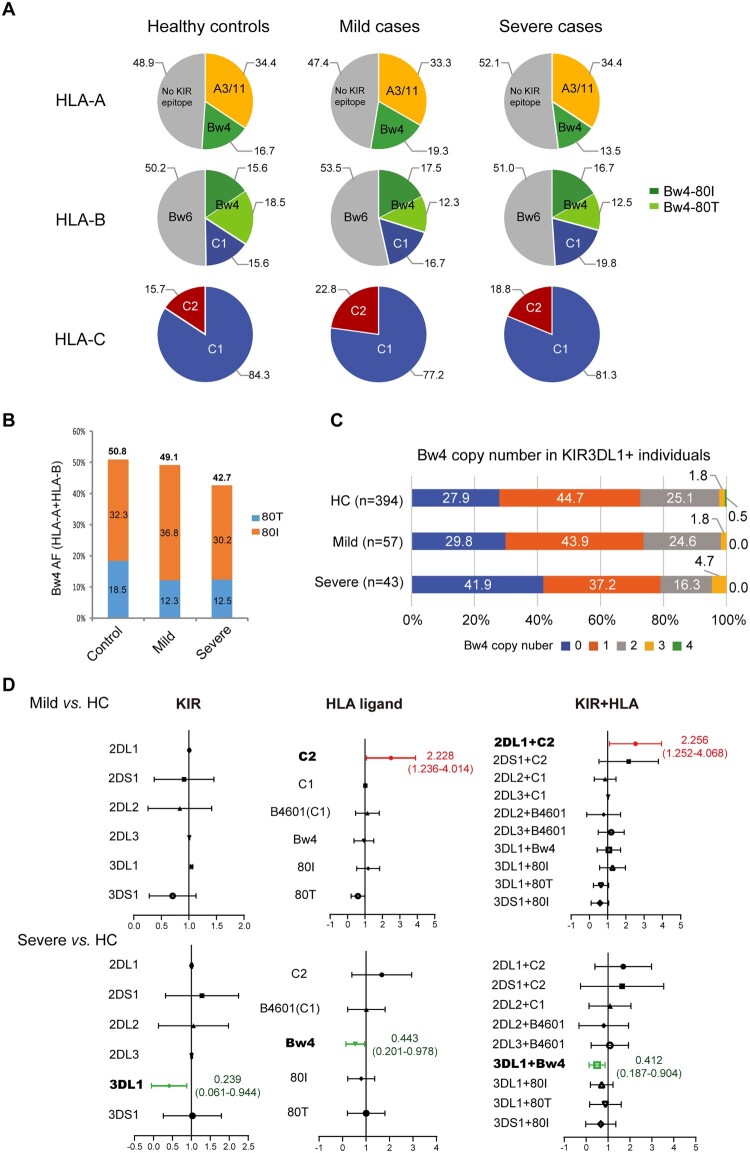
**Distributions of four epitopes of HLA-A, HLA-B and HLA-C that interact with human killer cell immunoglobulin-like receptors (KIRs) in Chinese healthy controls and COVID-19 patients. A**) Pie charts of the distributions of HLA class I epitopes that interact with KIRs in healthy controls and COVID-19 patients with mild or severe illness. The Bw4 epitope (residues 77, 80-83) is present in some HLA-A and HLA-B allotypes. Bw4 motifs are further divided into 80I (isoleucine; dark green) and 80 T (threonine; light green) motifs according to the amino acid at position 80. The C1 motif carried by HLA-B allotypes was introduced by B*46:01 in Asians. Allotypes that are not KIR ligands are in gray. **B**) Reduced Bw4-80I and Bw4-80 T at HLA-A and HLA-B allotypes in COVID-19 severe patients. **C**) Copy numbers of the Bw4 motif present at HLA-A and HLA-B (B-Bw4-80 T, B-Bw4-80I and A-Bw4) allotypes in KIR3DL1-positive individuals. **D**) Associations of KIR, HLA ligand genotypes and KIR + HLA combinations in mild or severe COVID-19. Odds ratios (ORs) and 95% confidence intervals (CIs) are shown.

Next, we compared the frequencies of HLA class I alleles that serve as ligands for NK cell receptors. HLA-A, -B, and -C allotypes were stratified according to their abilities to interact with KIRs. At HLA-A, the frequencies of HLA-A3/11 were similar between patients and controls. However, the prevalence of the HLA-A-Bw4 motif was lower in severe COVID-19 (13.5%) than in mild cases (19.3%) and controls (16.7%), but the difference was not significant. At HLA-B, the Bw4 motif was also reduced, and the C1 motif introduced by B*46:01 was increased in severe COVID-19. At HLA-C, which is expressed at much lower levels than HLA-A and HLA-B and is believed to be more specific for NK cell regulation rather than CTL activation, the distribution of HLA-C1 and HLA-C2 was similar between controls (C2: 15.7%) and severe COVID-19 patients (C2: 18.8%), but the C2 ligand was more frequent in mild COVID-19 patients (22.8%) (Figure 4A).

Overall, the most significant changes in the distribution of HLA-I ligands were the reduced HLA-Bw4 in the severe group, especially A-Bw4 and B-Bw4-80 T. At HLA-A and HLA-B, the allele frequencies of 80I and 80 T were decreased in severe COVID-19, while mild patients had HLA-Bw4 frequencies similar to those of controls but carried more 80I and less 80 T than controls (Figure 4B), which could be explained by the overall superior peptide-binding capacities of 80I than 80 T alleles (Figure 1E). At the genotypic level, more individuals lacked the Bw4 motif at HLA-A and HLA-B loci in severe patients (41.7%) than in controls (28.1%, *p *= 0.051). More C2 carriers were found in mild COVID-19 patients (45.6%) than controls (29.5%) (OR = 2.228, 95% CI = 1.236-4.014, *p *= 0.014), but the C2 genotype in severe patients (33.33%) was similar to controls (**Table S5**), and Bw4 + carriers were less represented in severe COVID-19 patients (HC, mild and severe cases: 71.9%, 70.2% and 58.2%) (**Table S6**). In addition, the copy number of the Bw4 motif in KIR3DL1-positive individuals also revealed that more individuals carried zero Bw4 copies in the severe patient group than in the other two groups (Figure 3C). These data indicated that the lack of HLA-Bw4 alleles might be related to worse outcomes of COVID-19.

The interaction between KIRs and cognate HLA ligands sets the threshold of NK cell activation. We evaluated the combination of KIRs with HLA genes encoding their respective ligands in the same individual. The frequencies of KIRs as HLA receptors were largely similar between patients and controls, except KIR3DL1 was underrepresented in severe cases, but the difference did not reach statistical significance (HC vs. severe: 95.4% vs. 89.6%, *p* = 0.170) (**Table S7**), and its ligand Bw4 allotypes (HLA-A and -B) were also decreased in severe COVID-19 but did not reach statistically significant (HC vs. severe: 71.9% vs. 58.3%, *p *= 0.051) (**Table S6**). However, examination of KIRs in the context of their cognate HLA ligands revealed a protective effect for KIR3DL1 in combination with HLA alleles bearing the Bw4 motif and KIR2DL1 in combination with HLA-C2 alleles. The KIR3DL1 + Bw4 combination was significantly reduced in severe patients compared with controls (52.1% vs. 68.8%, OR = 0.412, 95% CI = 0.187-0.904, *p *= 0.020) but was not changed in patients with mild illness (70.2%, *p* = 0.829). In contrast, the KIR2DL1 + C2 combination was overrepresented in the mild group (45.6% vs. 29.3%, OR = 2.256, 95% CI = 1.252-4.068, *p *= 0.013), but a difference in the frequency of the KIR2DL1 + C2 pair was not observed in the severe COVID-19 group (33.3%, *p *= 0.563) (**Table S8** and Figure 4D).

KIR3DL1 + Bw4 and KIR2DL1 + C2 are strong KIR-HLA pairs compared to other inhibitory KIR/HLA interactions [16, 17]. According to the “missing self” hypothesis, NK cells from strong inhibitory KIR/HLA carriers have superior functional potential upon stimulation with “missing self” cells expressing reduced HLA and the ability to inhibit viral replication. However, in contrast to HLA-C with 10-fold lower expression and a less efficient peptide-binding pocket compared to HLA-A and HLA-B, using Bw4 at HLA-A and HLA-B as major ligands for the inhibitory KIR comes at the cost of reducing peptide presentation when downregulated, unless the HLA ligands are not able to present viral peptides. Nonetheless, the reduced KIR3DL1/Bw4 pairs in severe patients and increased KIR2DL1/C2 pairs in mild patients indicate that the potency of NK cell education influences COVID-19 vulnerability.

### Downregulation of HLA class I by SARS-CoV-2 ORF8, but not the SARS-CoV-1 ortholog, may render cells susceptible to NK cell-mediated killing in COVID-19

**Figure 5.**
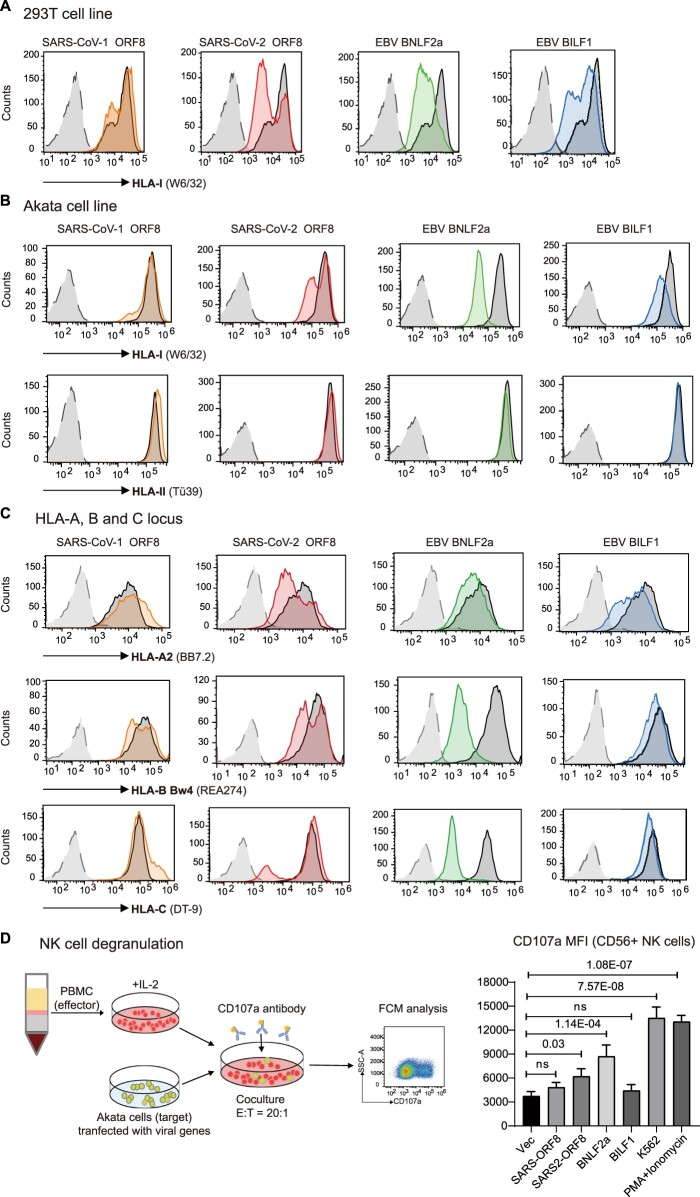
**SARS-CoV-2 ORF8 downregulates surface HLA-I and activates NK cells. A**) 293 T or **B**) Akata Burkitt lymphoma cells were transfected with pCDH-copGFP plasmids encoding different viral immune evasion genes. At 48 h posttransfection, surface HLA-I was stained with APC-conjugated W6/32 mAb, and HLA-II was stained with PE-conjugated Tü39 mAb (in Akata only) gated on GFP^+^ cells. Black: transfected with vector, orange: transfected with SARS-CoV ORF8, red: transfected with SARS-CoV2 ORF8, green: transfected with EBV BNLF2a, and blue: transfected with EBV BILF1. The gray histogram denotes background staining obtained with an isotype control antibody. **C**) Downregulation of HLA-A, -B and -C by viral proteins. Flow cytometry staining of HLA-A (A2), HLA-B (Bw4) or HLA-C using the locus-specific mAbs is shown for transfected cells and an isotype control (grey). 293 T cells were selected to detect the regulation on HLA-A2, Akata cells were used to detect the regulation on HLA-B Bw4 and HLA-C. **D**) NK cells activation determined by NK cells degranulation assay. Akata as target cells were incubated with PBMCs of a representative healthy donor as effector cells, both target and effector cells were heterogenous for HLA-A Bw4 80I and HLA-B Bw4-80I, and homozygous for HLA-C1. Flow cytometry determined CD107a expression on the CD56^+^ cell surfaces following stimulation of Akata cells transfected with empty vector, ORF8 of SARS-CoV-1 and −2, EBV proteins BNLF2a and BILF1, or with K562 cells, or with PMA/ionomycin (E:T ratio 20:1). The histogram shows the mean fluorescence intensity (MFI) of surface CD107a expression on NK cells (gated on CD56^+^/ CD19^-^). Representative data are shown from three independent experiments, statistical significance was assessed by one-way ANOVA with Dunnett t multiple-comparison test. Data are depicted as the mean ± s.d.

Downregulation of cell surface HLA class I is a prerequisite for recognition and elimination of virus-infected cells by NK cells. It has been reported that actively replicating SARS-CoV-2 causes HLA-I downregulation through lysosomal degradation by the accessory protein open reading frame 8 (ORF8) [14]. ORF8 in SARS-CoV-2 and only shares 40% amino acid identity with SARS-CoV-1 ORF8. To compare the immune evasion activities of SARS-CoV-2 and a ubiquitous human herpesvirus EBV, plasmids encoding ORF8 proteins of SARS-CoV-1 and SARS-CoV-2 and BNLF2a and BILF1, two well-known immune evasion proteins of EBV, were transfected into 293 T and Akata cell lines. Consistent with previous reports [14, 30–32], ectopic expression of SARS-CoV-2 ORF8, but not the homolog of SARS-CoV-1, downregulated surface HLA-I expression in both 293 T and Akata cells. The extent of HLA-I downregulation by SARS-CoV-2 ORF8 was comparable to that mediated by EBV BILF1, which also targets HLA-I for degradation via lysosomes [32], and to a greater extent, EBV BNLF2a, which prevents the binding of both peptides and ATP to TAP [30] (Figure 5A). However, none of these viral immune evasion proteins affected HLA class II levels at the cell surface, as demonstrated in Akata cells that expressed HLA class II (Figure 5B).

We then asked whether SARS-CoV-2 ORF8 downregulates all of the three HLA-I loci, HLA-A, -B and -C. Using allotype-specific monoclonal antibodies, SARS-CoV-2 ORF8 was found to be able to downregulate HLA-A, -B and -C molecules, while EBV BILF1 only downregulated HLA-A but exhibited negligible downregulation of HLA-B/C. In contrast to BILF1, EBV BNLF2a showed the greatest reduction magnitude in HLA-B/C, and to a lesser extent in HLA-A downregulation. And consistently, SARS-CoV-1 ORF8 was unable to downregulate any HLA-I locus **(**Figure 5C**)**. Differential downregulation of HLA-I at three loci might represent different viral immune evasion mechanisms.

Loss of HLA can trigger NK cell-mediated lysis of the infected cells because of the failure of HLA ligand to bind inhibitory KIRs. NK cells activation by target cells expressing SARS-CoV-2 ORF8 and EBV immune evasion proteins were determined by NK cell degranulation assay [33]. Freshly isolated PBMCs from healthy donor were incubated with Akata cells (both donor and Akata cells possess HLA-A Bw4, -B Bw4 and -C1 genotype) transfected with different viral proteins, or MHC devoid K562 cells, or PMA/ionomycin stimulation. Following incubation in the presence of CD107a antibody, the surface level of CD107a on CD56^+^ NK cells was determined by flow cytometry. The results suggested that SARS-CoV-2 expression in target cells could activate NK cells. CD107a expression on the surface of NK cells increased 1.7-fold by co-incubation with Akata cells transfected with SARS-CoV-2 ORF8, 2.3-fold increase by transfection of EBV BNLF2, and the positive control, HLA devoid cell line K562 caused 3.6-fold increase on surface CD107a. On the contrary, target cells expressing SARS-CoV-1 ORF8 or EBV BILF1 did not cause appreciable activation of NK cells (Figure 5D). The effects of NK cell activation by these viral immune evasion proteins were largely consistent with the extent of HLA-I downregulation by each viral protein.

The association of HLA-Bw4/KIR3DL1 in severe but not mild COVID-19 cases may be explained by the differential MHC-Ι expression in the infected individuals with high or low viral loads. Using a mouse model, it has been shown that only the replicating group, not the recovered group, displayed MHC-I downregulation after SARS-CoV-2 infection [14]. Indeed, acute infection with SARS-CoV-2 stimulates MHC-I expression, while upon viral replication, ORF8 synthesis is increased to degrade the cell surface MHC-I that results in CTL evasion [14]. Therefore, NK cell-mediated cytotoxicity may be more important for severe cases with active SARS-CoV-2 replication and high viral load but did not play a significant role in mild patients with low viral titres, and the infected cells were predominantly cleared by HLA-I-mediated CTLs.

### Validation of HLA-Bw4 in Russian COVID-19 patients

**Figure 6.**
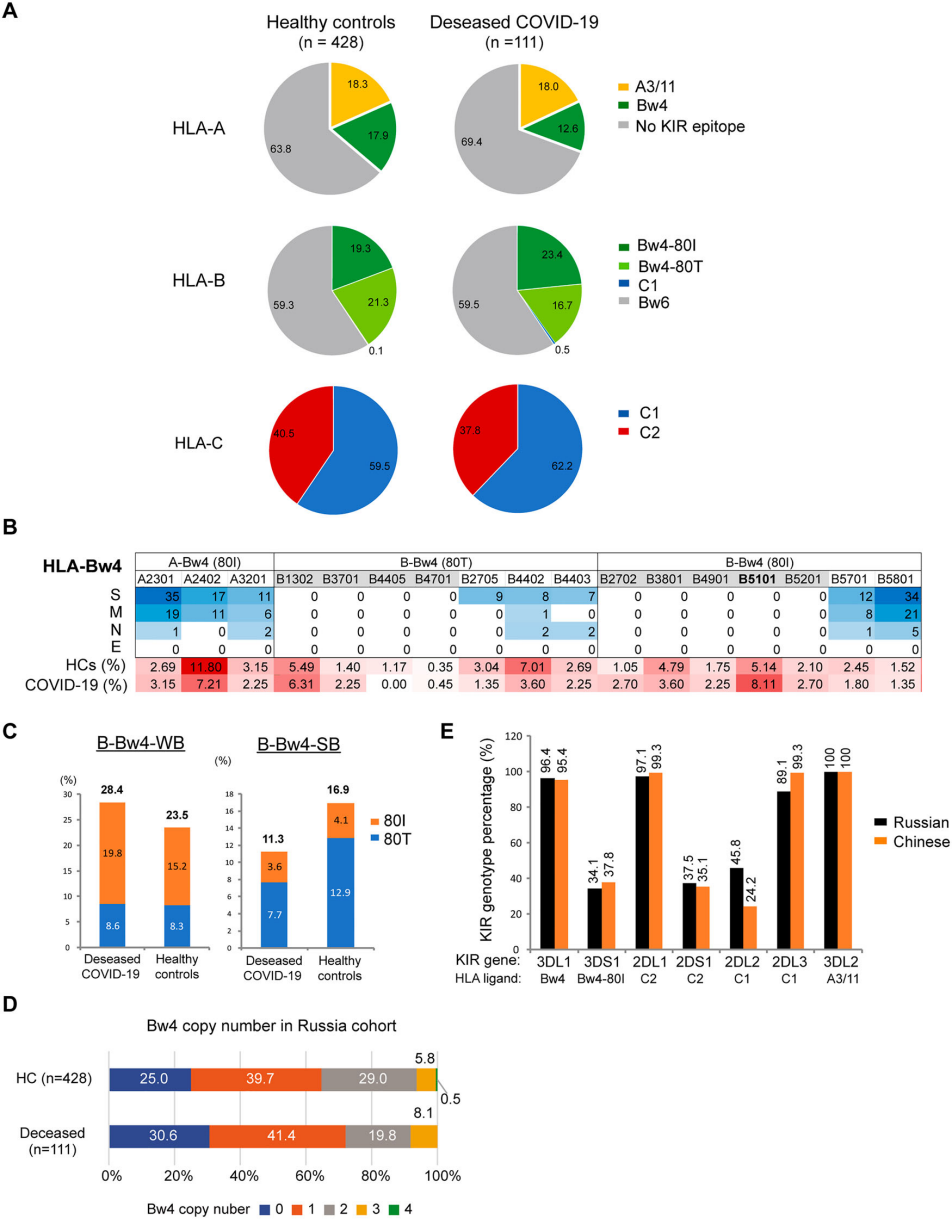
**Distribution of the HLA-Bw4 motif in healthy controls and deceased COVID-19 patients in Russia** [10]**. A**) Pie charts of the distributions of HLA class I epitopes that interact with KIRs in Russian healthy controls (n = 428) and deceased COVID-19 patients (n = 111). The source data were from [10]. **B**) Allele frequencies of the Bw4 motif in HLA-A and HLA-B allotypes in controls and deceased COVID-19 patients in Russia. **C**) Distributions of weak binder (WB) and strong binder (SB) of HLA-B and HLA-A alleles in controls and deceased COVID-19 patients. **D**) Copy numbers of the Bw4 motif present at HLA-A and HLA-B alleles (B-Bw4-80 T, B-Bw4-80I and A-Bw4) in Russian controls and deceased COVID-19 patients. **E**) Percentages of KIR genes as receptors for HLA-Bw4, HLA-C1 and C2 allotypes in Russians and Chinese. Data of Russian KIR gene frequencies were pooled from 6 populations in Russia with a total of 384 individuals from www.allelefrequencies.net. Chinese KIR gene frequencies were from this study (n = 413).

To overcome the small sample size and limited generalizability of our study, we tried to validate the association of HLA with COVID-19 development in a different ethnic population. One study from Russia shared the HLA genotypes of individual healthy controls (n = 428) and deceased COVID-19 patients (n = 111) [10], which enabled further analysis based on the KIR-binding characteristics of HLA class I allotypes. Similar to our Chinese cohort, the most noticeable differences in HLA motif distributions of the Russian cohort were HLA-A-Bw4 and HLA-B-Bw4-80 T motifs. The allele frequency of HLA-A-Bw4 was reduced in the deceased Russian COVID-19 patients compared to their controls (18.5% vs. 22.1%); although the overall frequency of HLA-Bw4 allotypes was similar in patients and controls, HLA-B-Bw4-80 T alleles were reduced in the deceased patients (16.7%) compared to controls (21.3%), while HLA-B-Bw4-80I was increased in COVID-19 (23.4%) than controls (19.3%) (Figure 6A).

Most HLA-B-Bw4-80I endemic in Russia were weak binders for SARS-CoV-2 peptides by prediction, while HLA-B-Bw4-80 T in Russia was enriched with strong binders (Figure 6B), which was in contrast to Chinese, whose weak binders of HLA-B-Bw4 were mostly classified as the 80 T allotype (Figure 2C). The deceased patients had a lower incidence of HLA-B-Bw4 alleles as strong binders (SB) than controls (11.3% vs. 16.9%) and a slightly higher incidence of Bw4-weak binders than controls at HLA-B (28.4% vs. 23.5%) (Figure 6C), suggesting an important role of CD8+ T-cell-mediated killing in the protection of SARS-CoV-2 mortality in the Russian population.

The reduction in Bw4 copy number in Russian patients was not as evident as in Chinese severe patients. The percentages of individuals who did not carry any Bw4 motif were 30.6% in patients and 25% in controls (Figure 6D). Instead, the reduction in HLA alleles with strong affinities for viral peptides was more profound in deceased Russian patients than in Chinese severe patients (Russian: 16.9% vs. 11.3%, Chinese: 9.1% vs. 8.3%, Figure 3B). Russians and Chinese had similar frequencies of KIR genes (Figure 6E). It seems that NK-mediated anti-SARS-CoV-2 activity was less important than T-cell killing in the Russian population. Therefore, the mild protective roles of HLA-A-Bw4 and HLA-B-Bw4-80 T in Russian patients might largely result from the superior antigen-presenting abilities of these alleles to CTLs rather than NK cell activation.

### Distribution of HLA-Bw4 allotypes in world populations and their binding affinities for SARS-CoV-2 peptides

**Figure 7.**
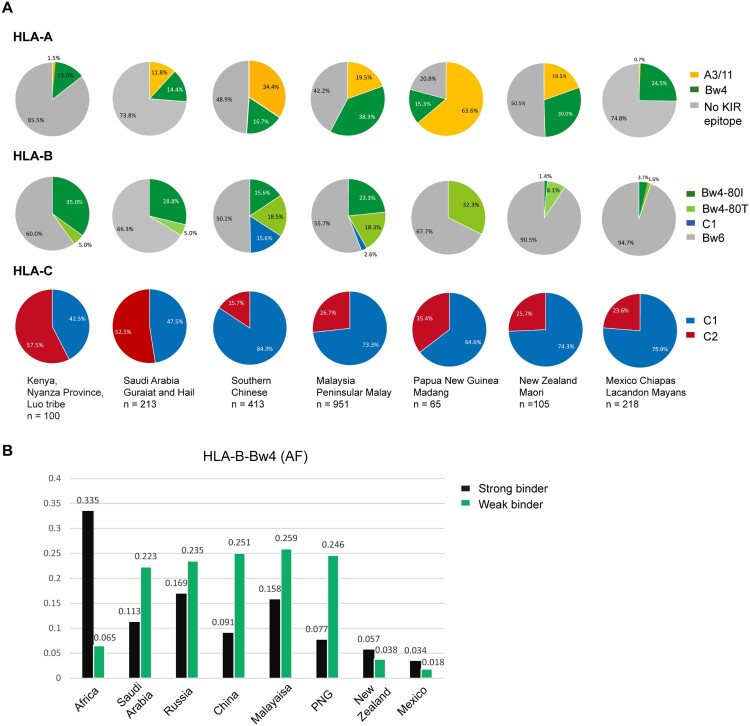
**HLA class I as KIR ligands in different populations. A**) Pie charts showing the frequency spectra for HLA class I alleles that interact with KIRs in different ethnic populations. The A3/11 epitope is carried by HLA-A3 and HLA-A11; the Bw4 epitope is encoded by subsets of HLA-A and HLA-B alleles; and the C1 epitope present in HLA-B is carried by HLA-B*46 and HLA-B*73. Gray pie segments represent allotypes that are not KIR ligands. N denotes the population size. **B**) Percentages of HLA-B alleles carrying the Bw4 motif that are predicted to interact with SARS-CoV-2 peptides with strong or weak affinities. Data of non-Chinese populations except Russia (n = 428) [10] were from http://allelefrequencies.net/. The prevalence of HLA-Bw4 allotypes as poor binders for coronaviruses in certain areas might be a result of historical competition between NK- and T-cell-mediated immunity against epidemics of infectious diseases.

It has been suggested that East Asians are enriched for interactions between inhibitory KIR and HLA-A and HLA-B compared with other populations [34]. To gain insight into whether NK cell-mediated anti-SARS-CoV-2 immunity is particularly important for East Asians, we analyzed HLA-A, -B and -C, which act as KIR ligands in distinct ethnic populations. The results suggested that African and East Asians carried the most abundant Bw4 allotypes at HLA-B; in contrast, the New World populations, such as indigenous people in New Zealand and Mexico, carried the least HLA-B-Bw4. Besides, East Asians carried the fewest C2 motifs at HLA-C (Figure 7A).

In addition to Bw4 frequency, the binding affinities for SARS-CoV-2 peptides of HLA-B allotypes encoding the Bw4 motif vary significantly between populations. Most HLA-B-Bw4 encoded by Sub-Saharan Africans had strong affinities for SARS-CoV-2 peptides, while most HLA-B-Bw4 alleles in East Asia were poor binders for SARS-CoV-2 peptides (Figure 7B). The disadvantage of the restrictive SARS-CoV-2 peptide repertoire of the HLA-Bw4 weak binder group may be overridden by NK cell licensing during viral replication, but when viral titres were low, limited ORF8 expression did not cause obvious MHC-I downregulation, and MHC alleles with higher affinity for SARS-CoV-2 peptides played a superior protective role in the infected individuals. More functional studies are needed to directly address the involvement of T- and NK-cell-mediated anti-SARS-CoV-2 immune responses in the whole process of viral infection and replication, and it will be of interest to elucidate the contribution of HLA-I/viral peptide-mediated CTL and HLA-I/KIR-mediated NK cell licensing to COVID-19 outcomes in different ethnic populations.

## Discussion

Human genetic variations underlie differential susceptibility to infectious diseases. Here, in-depth analysis of the HLA and KIR genotypes and their interaction system in Chinese COVID-19 patients with different clinical presentations supports the notion that in addition to T-cell-mediated immunity, NK cell-mediated immune defense also plays a critical role in the anti-SARS-CoV-2 response, at least in an Asian population with a high number of detrimental HLA class I alleles in binding coronavirus peptides but with enhanced NK cell repertoires.

HLA class I has dual roles in the immune response by presenting peptides to CTLs and educating NK cells through interactions with KIRs. The engagement of inhibitory receptors on NK cells by self-HLA class I molecules is the key event that determines whether an NK cell will be functionally capable of mediating missing-self recognition (education) or whether it will be hyporesponsive following stimulation. The HLA genotype that acts as a KIR ligand varies significantly in different populations. In our Chinese COVID-19 patients, inhibitory KIR/HLA pairs were found to be protective against COVID-19 progression. Lack of KIR3DL1 + Bw4 resulted in an increased risk of severe disease, whereas KIR2DL1 + C2 was associated with mild symptoms. The association of these two pairs with COVID-19 severity might be due to their superior binding strength in the iKIR/HLA interaction system, and differential regulation by ORF8. The Bw4 motif is present in some HLA-A and HLA-B allotypes, and the protein levels of HLA-A and HLA-B are ∼10-fold higher than those of HLA-C [35]. Therefore, the KIR3DL1 and HLA-Bw4 interaction may play a vital role in NK cell licensing, and loss of this strong interaction may underlie the increased risk of developing severe COVID-19. A similar scenario has been reported in HIV disease progression [36]. On the other side, the reduction magnitude in HLA-C locus seems to be greater than that of HLA-A/B by SARS-CoV-2 ORF8 (Figure 5C). It is likely that the relatively low level of ORF8 is able to downregulate HLA-C, but not HLA-A/B, upon mild infection. Among KIR/HLA-C pairs, inhibitory KIR2DL1 binds to C2 with higher affinity than KIR2DL2/3 to C1, which may account for the increased KIR2DL1 + C2 combination in mild, but not severe COVID-19 patients. It is conceivable that NK cells educated by strong iKIR/HLA combinations were superior effectors against missing-self cells infected with SARS-CoV-2.

**Figure 8.**
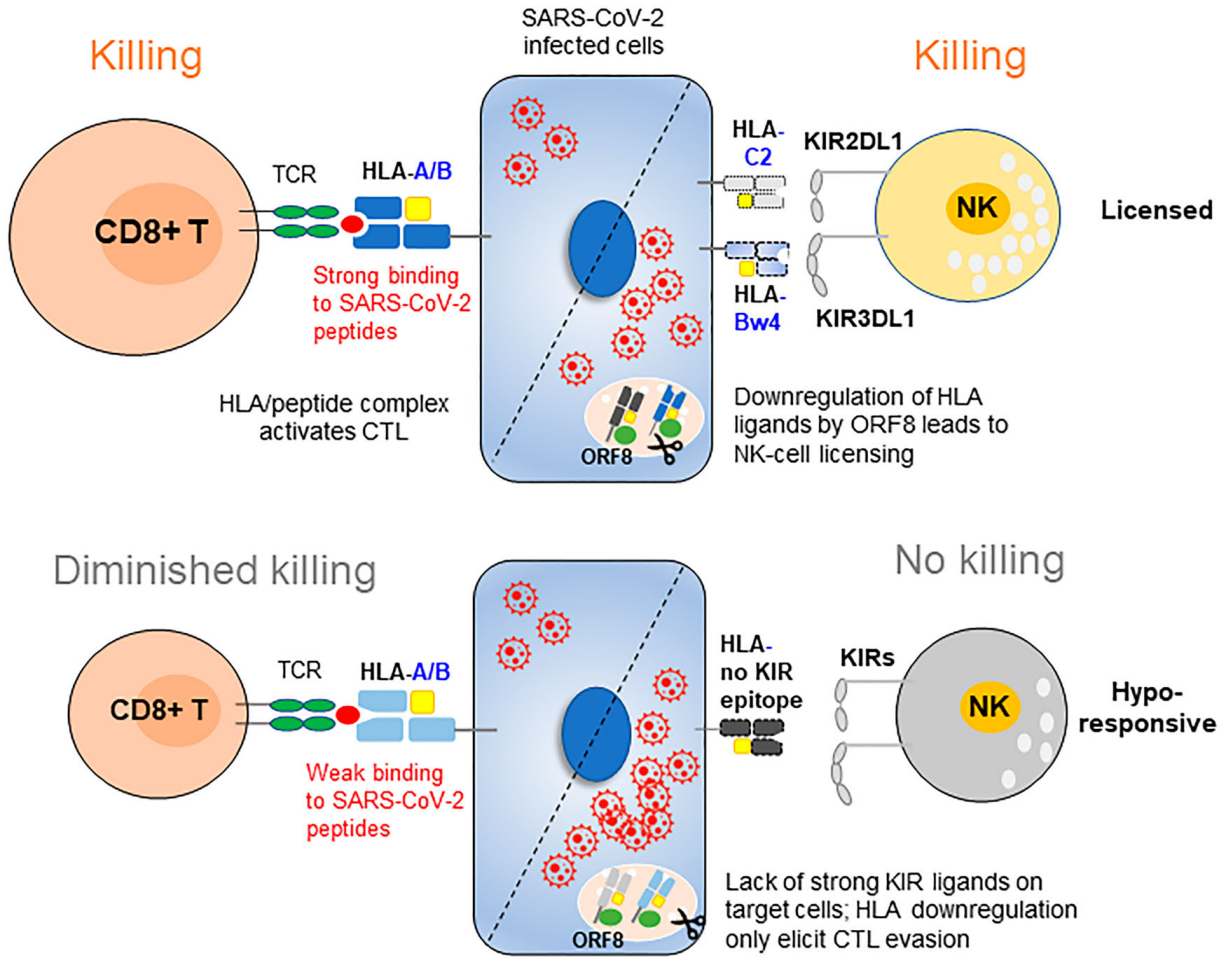
**Working model**. In individuals with immune protection against SARS-CoV-2, CTLs play a dominant role in eliminating viral infection. When the viral load is low and HLA-I is normally expressed, CTLs play a dominant role in destroying the infected cells; upon viral replication and SARS-CoV-2 ORF8-induced HLA-I degradation, the strong interaction of HLA-C2 ligands and KIR2DL1 promotes NK cell activation. In contrast, in individuals who are prone to develop severe symptoms, their HLA genotypes did not have viral peptide presentation advantages, and their NK cells are missing inhibitory KIR3DL1 and HLA-Bw4 interactions and are hyporesponsive against the high titre of SARS-CoV-2, which causes HLA-I degradation and CTL evasion.

HLA-A/B are dominant CTL ligands throughout infection. In cases where HLA-A/B is the cognate-ligand for inhibitory KIR, SARS-CoV-2 ORF8-mediated downregulation of HLA-I ligands may actually enhance NK-cell control but evade CTL killing of viral infection. The peptide affinities of these HLA ligands determine the balance between CTL evasion and NK cell activation. If HLA-I alleles are strong binders for SARS-CoV-2 peptides but not ligands for NK cells, such as B*35:01 and B*55:02, both belong to Bw6 epitope, the net effect of their downregulation might be immune evasion; if HLA-I alleles are poor binders for viral epitopes but as ligands for NK cell receptor, such as a subset of Bw4 alleles and HLA-B*46:01 which possess a C1 motif, their downregulation would not impair CTL but facilitate NK cell clearance of SARS-CoV-2-infected targets (Figure 8). Most weak coronavirus binders common in Southern Chinese belong to HLA-B-Bw4-80 T, such as B*13:01, B*27:04, B*37:01 and B38:02, while in Russians, most WBs are classified as HLA-B-Bw4-80I allotypes. Utilizing 80 T rather than 80I as poor binders for viral peptides may be more beneficial for infected individuals because Bw4-80I is also able to interact with the activating receptor KIR3DS1, but Bw4-80 T does not bind to KIR3DS1, and ligation to inhibitory KIRs might be compensated for by the interaction with activating KIRs. Indeed, a lower frequency of KIR3DS1 was found in the mild COVID-19 group but not in the severe group, and a reduction in KIR3DL1 incidence was observed only in severe COVID-19 (**Table S7**). In this regard, East Asians are most likely to benefit from NK cell education/licensing during SARS-CoV-2 infection who carry the most abundant poor binders at HLA-B (Bw4-WB: 25.1%, B*46:01: 15.6%, total WBs is 40.7% in HLA-B allotypes, highest in populations as shown in Figure 7) and fewest activating KIRs than other populations [37].

Limitations of this work exist. First, our analysis was restricted to individuals of Chinese ancestry, which limits the generalizability of our findings to diverse ethnic groups. Second, we were unable to conduct formal statistical replication in an independent sample due to the lack of sample availability. Third, asymptomatic COVID-19 carriers are better controls than the non-infected healthy individuals. Nevertheless, our successful replication of several previously reported HLA alleles associated with COVID-19 supports the credibility of our work, and HLA-I downregulation and NK cell activation by SARS-CoV-2 ORF8 also suggests a potential role of HLA/KIR-mediated NK cell activation in COVID-19 outcome.

In conclusion, this study provides genetic evidence that inhibitory KIRs with HLA class I ligands, especially HLA-Bw4/KIR3DL1 interactions, exert protective effects against severe COVID-19 in a Chinese population. Considering the genetic heterogeneity in different ethnic groups, larger cohorts across different geographies and ethnicities, together with functional studies, should be conducted to obtain conclusive evidence of the association between the HLA/KIR interaction system in the immune response and disease progression of COVID-19 and to dissect the role of CTL- and NK cell-mediated killing in the process of SARS-CoV-2 infection and replication.

## Author contributions

LF, JZ and YXZ are the principal investigators of this study. LF, JZ, YXZ, RW, YS, BHK conceived and designed all the experiments. RW, YS, BHK performed the experiments. RW, YS, BHK, JL, XY, JT, YL, YXL, JZ, TC collected samples. RW, YS analyzed the data. XX, HZ provided plasmids. LF and RW wrote the manuscript. All authors contributed to the article and approved the submitted version.

## Supplementary Material

Supplemental Material

## Data Availability

We are happy to share reagents and information in this study upon request.
