# Supplementary material for: HLA-Bw4 in association with KIR3DL1 favors natural killer cell-mediated protection against severe COVID-19
Source: Emerg Microbes Infect. 2023 Mar 13;12(1):2185467. doi: 10.1080/22221751.2023.2185467 (PMC10013568; doi:10.1080/22221751.2023.2185467)
Supplement: Supplemental Material [file TEMI_A_2185467_SM7602.zip › Supplementary Table 2_8.docx]

**Table** **S2** Characteristics of healthy control and COVID-19 patients

| Group | Healthy controls  （n = 413） | COVID-19  patients  (n=105) | *P-* value |
| --- | --- | --- | --- |
|  |  |  |  |
| Age  Median (range) | 36 (16-78) | 50 (23-84) | **2.374E-17** |
| Sex |  |  |  |
| Male | 219 (53.0%) | 63 (60.0%) | 0.200 |
| Female | 194 (47.0%) | 42 (40.0%) |  |
| Geographic area |  |  |  |
| Southern China | 375 (90.8%) | 96 (91.4%) | 0.841 |
| Northern China | 38 (9.2%) | 9 (8.6%) |  |
| Hypertension | 4 (1.0%) | 19 (18.1%) | **2.101E-13** |
| Coronary artery disease | 0 (0) | 4 (3.8%) | **0.002** |
| Diabetes | 2 (0.5%) | 13 (12.4%) | **7.030E-10** |

The t-test was used to compare the mean age of severe and mild patients, and the Pearson chi-square (χ^2^) test, continuity correction (when the number of subjects in a cell is <5) or Fisher’ exact test (when the number of subjects in a cell is <1) was used to compare the frequency of sex, hypertension, coronary artery disease and diabetes in severe and mild patients. Bold values indicate *P* < 0.05. Northern and southern region of China are divided by the Yangtze River.

**Table S3** Characteristics of COVID-19 patients with severe or mild symptoms

| Group | Severe cases  (n = 48) | Mild cases  (n = 57) | *P* |
| --- | --- | --- | --- |
| Age  Median (range) | 56 (26-84) | 45 (23-77) | **3.450E-4** |
| Sex |  |  |  |
| Male | 32 (66.7%) | 31 (54.4%) | 0.201 |
| Female | 16 (33.3%) | 26 (45.6%) |  |
| Hypertension | 16 (33.3%) | 3 (5.5%) | **1.980E-4** |
| Coronary artery disease | 4 (8.3%) | 0 (0%) | 0.041 |
| Diabetes | 11 (22.9%) | 2 (3.5%) | **0.003** |

The t-test was used to compare the mean age of severe and mild patients, and the Pearson chi-square (χ2) test, continuity correction (when the number of subjects in a cell is <5) or Fisher’ exact test (when the number of subjects in a cell is <1) was used to compare the frequency of sex, hypertension, coronary artery disease and diabetes in severe and mild patients. Bold values indicate P < 0.05.

**Table S4** The frequency and odds ratio values of the HLA-I alleles in healthy control and COVID-19 patients

| Allele^a^ | HC  (n=413) % | Mild case  (n=57)  % | Severe case  (n=48)  % | Mild vs. HC | | Severe vs. HC | |
| --- | --- | --- | --- | --- | --- | --- | --- |
|  |  |  |  | *OR*  (95% *CI*) | *P-* value | *OR*  (95% *CI*) | *P-* value |
| **HLA-A** |  |  |  |  |  |  |  |
| A*01:01 | 1.7 | 1.8 | 0.0 |  | 1.000 |  | 0.398 |
| **A*02:07** | 9.8 | 14.9 | 17.7 |  | 0.094 | **2.003**  **(0.857-4.678)** | **0.017** |
| A*02:01 | 9.1 | 8.8 | 8.3 |  | 0.914 |  | 0.809 |
| A*02:03 | 6.4 | 4.4 | 7.3 |  | 0.524 |  | 0.742 |
| A*02:06 | 4.2 | 3.5 | 2.1 |  | 0.908 |  | 0.457 |
| A*11:01 | 29.8 | 29.8 | 29.2 |  | 0.993 |  | 0.901 |
| A*11:02 | 3.4 | 3.5 | 2.1 |  | 1.000 |  | 0.705 |
| A*03:01 | 1.1 | 0.0 | 3.1 |  | 0.544 |  | 0.234 |
| A*24:02 | 14.4 | 16.7 | 10.4 |  | 0.523 |  | 0.286 |
| A*26:01 | 2.3 | 0.00 | 5.2 |  | 0.200 |  | 0.175 |
| A*29:01 | 1.3 | 0.00 | 0.0 |  | 0.438 |  | 0.522 |
| A*30:01 | 2.9 | 2.6 | 3.1 |  | 1.000 |  | 1.000 |
| A*31:01 | 1.9 | 4.4 | 1.0 |  | 0.187 |  | 0.829 |
| A*33:03 | 8.8 | 7.0 | 6.3 |  | 0.516 |  | 0.391 |
| **HLA-B** |  |  |  |  |  |  |  |
| B*07:05 | 1.3 | 0.00 | 0.0 |  | 0.438 |  | 0.522 |
| B*13:01 | 6.4 | 4.4 | 2.1 |  | 0.398 |  | 0.090 |
| B*13:02 | 2.9 | 3.5 | 3.1 |  | 0.951 |  | 1.000 |
| B*15:01 | 3.4 | 3.5 | 5.2 |  | 1.000 |  | 0.537 |
| B*15:02 | 5.8 | 7.0 | 3.1 |  | 0.610 |  | 0.276 |
| B*27:04 | 1.3 | 0.9 | 1.0 |  | 1.000 |  | 1.000 |
| **B*35:01** | 1.7 | 5.3 | 3.1 | **3.428**  **(1.213-9.690)** | **0.033** |  | 0.559 |
| B*37:01 | 1.3 | 0.9 | 0.0 |  | 1.000 |  | 0.522 |
| B*38:02 | 4.7 | 0.9 | 3.1 |  | 0.097 |  | 0.652 |
| B*39:01 | 2.1 | 0.9 | 4.2 |  | 0.619 |  | 0.342 |
| B*40:01 | 15.5 | 12.3 | 14.6 |  | 0.369 |  | 0.815 |
| B*40:02 | 1.7 | 0.0 | 1.0 |  | 0.323 |  | 0.958 |
| B*40:06 | 1.6 | 2.6 | 0.0 |  | 0.666 |  | 0.435 |
| B*44:03 | 1.1 | 1.8 | 1.0 |  | 0.877 |  | 1.000 |
| B*46:01 | 15.6 | 16.7 | 19.8 |  | 0.773 |  | 0.292 |
| B*48:01 | 1.1 | 0.9 | 0.0 |  | 1.000 |  | 0.632 |
| **B*51:01** | 4.1 | 6.1 | 9.4 |  | 0.455 | **2.631**  **(0.901-7.685)** | **0.040** |
| B*51:02 | 1.5 | 1.8 | 0.0 |  | 1.000 |  | 0.476 |
| B*52:01 | 1.8 | 0.0 | 2.1 |  | 0.293 |  | 1.000 |
| B*54:01 | 2.4 | 2.6 | 1.0 |  | 1.000 |  | 0.620 |
| **B*55:02** | 2.9 | 7.0 | 2.1 | **2.506**  **(1.036-6.066)** | **0.046** |  | 0.893 |
| B*58:01 | 7.1 | 7.0 | 5.2 |  | 0.961 |  | 0.480 |
| **HLA-C** |  |  |  |  |  |  |  |
| C*01:02 | 20.5 | 18.4 | 25.0 |  | 0.611 |  | 0.301 |
| C*03:02 | 6.8 | 7.0 | 5.2 |  | 0.925 |  | 0.558 |
| C*03:03 | 4.7 | 3.5 | 8.3 |  | 0.597 |  | 0.179 |
| C*03:04 | 13.1 | 11.4 | 8.3 |  | 0.617 |  | 0.185 |
| C*04:01 | 4.4 | 3.5 | 6.3 |  | 0.862 |  | 0.560 |
| C*04:03 | 1.6 | 2.6 | 2.1 |  | 0.666 |  | 1.000 |
| C*06:02 | 4.6 | 7.9 | 4.2 |  | 0.130 |  | 1.000 |
| C*07:02 | 18.5 | 11.4 | 19.8 |  | 0.062 |  | 0.763 |
| C*08:01 | 8.0 | 9.7 | 3.1 |  | 0.545 |  | 0.086 |
| C*12:02 | 2.8 | 0.9 | 2.1 |  | 0.372 |  | 0.945 |
| C*12:03 | 2.7 | 3.5 | 2.1 |  | 0.833 |  | 1.000 |
| C*14:02 | 3.6 | 4.4 | 6.3 |  | 0.893 |  | 0.330 |
| C*15:02 | 2.8 | 6.1 | 2.1 |  | 0.104 |  | 0.945 |
| C*15:05 | 1.2 | 0.0 | 0.0 |  | 0.488 |  | 0.573 |

*P* values comparing cases and control are derived from using the Pearson chi-square (*χ*^2^) test, continuity correction (when the number of subjects in a cell is <5) or Fisher’ exact test (when the number of subjects in a cell is <1). All significant differences disappeared after Bonferroni correction.

CI = confidence interval, OR = odds ratio. ORs were computed and 95% CIs were calculated using logistic regression, adjusted for sex, age, hypertension, coronary artery disease and diabetes.

^a^Allele: only alleles with a frequency greater than 1% in healthy controls were included.

**Table S5** The frequency of the KIR ligands in healthy control and COVID-19 patients

| Ligands | Healthy control n = 413 | |  | Mild cases  n = 57 | |  | Severe cases  n = 48 | |  | *P-* value | |
| --- | --- | --- | --- | --- | --- | --- | --- | --- | --- | --- | --- |
|  | % | (N) |  | % | (N) |  | % | (N) |  | HC vs. Mild | HC vs. Severe |
| **HLA-A** |  |  |  |  |  |  |  |  |  |  |  |
| A3/11 | 34.4 | (284) |  | 33.3 | (38) |  | 34.4 | (33) |  | 0.825 | 0.999 |
| Bw4 | 16.7 | (138) |  | 19.3 | (22) |  | 13.5 | (13) |  | 0.490 | 0.428 |
| non-KIR ligand | 48.9 | (404) |  | 47.4 | (54) |  | 52.1 | (50) |  | 0.757 | 0.556 |
| **HLA-B** |  |  |  |  |  |  |  |  |  |  |  |
| C1 | 15.6 | (129) |  | 16.7 | (19) |  | 19.8 | (19) |  | 0.773 | 0.292 |
| Bw4^a^ | 34.1 | (282) |  | 29.8 | (34) |  | 29.2 | (28) |  | 0.361 | 0.329 |
| 80I | 15.6 | (129) |  | 17.5 | (20) |  | 16.7 | (16) |  | 0.598 | 0.789 |
| 80T | 18.5 | (153) |  | 12.3 | (14) |  | 12.5 | (12) |  | 0.102 | 0.145 |
| Bw6 | 50.2 | (415) |  | 53.5 | (61) |  | 51.0 | (49) |  | 0.513 | 0.882 |
| **HLA-C** |  |  |  |  |  |  |  |  |  |  |  |
| C1 | 84.3 | (696) |  | 77.2 | (88) |  | 81.3 | (78) |  | 0.057 | 0.447 |
| C2 | 15.7 | (130) |  | 22.8 | (26) |  | 18.8 | (18) |  | 0.057 | 0.447 |

P values comparing cases and control are derived from using the Pearson chi-square (χ2) test, continuity correction (when the number of subjects in a cell is <5) or Fisher’ exact test (when the number of subjects in a cell is <1).

^a^ The Bw4 motif here includes the HLA-B Bw4-80I and HLA-B Bw4-80T.

**Table S6** The frequency and odds ratio values of the HLA-I genotype in healthy control and COVID-19 patients

| Genotype | Healthy control  n = 413 | | Mild cases  n = 57 | | Severe cases  n = 48 | | Mild vs. HC | | Severe vs. HC | |
| --- | --- | --- | --- | --- | --- | --- | --- | --- | --- | --- |
|  | % | (N) | % | (N) | % | (N) | *OR*  (95% *CI*) | *P-* value | *OR*  (95% *CI*) | *P-* value |
| Individual with Bw4 | 71.9 | (297) | 70.2 | (40) | 58.3 | (28) |  | 0.785 |  | 0.051 |
| Individual with C1 | 98.1 | (405) | 100.0 | (57) | 95.8 | (46) |  | 0.608 |  | 0.631 |
| Individual with C2 | 29.5 | (122) | 45.6 | (26) | 33.3 | (16) | 2.228  (1.236-4.014) | **0.014** |  | 0.587 |

P values comparing cases and control are derived from using the Pearson chi-square (χ2) test, continuity correction (when the number of subjects in a cell is <5) or Fisher’ exact test (when the number of subjects in a cell is <1). CI = confidence interval, OR = odds ratio, NS = not significant. ORs were computed and 95% CIs were calculated using logistic regression, adjusted for sex, age, hypertension, coronary artery disease and diabetes.

**Table S7** KIR frequencies in controls and COVID-19 patients

| KIRs | Healthy control n = 413 | |  | Mild cases  n = 57 | |  | Severe cases  n = 48 | |  | *P-* value | |
| --- | --- | --- | --- | --- | --- | --- | --- | --- | --- | --- | --- |
|  | % | (N) |  | % | (N) |  | % | (N) |  | HC vs. Mild | HC vs. Severe |
| 3DL1 | 95.4 | (394) |  | 100.0 | (57) |  | 89.6 | (43) |  | 0.196 | 0.170 |
| 3DS1 | 37.8 | (156) |  | 28.1 | (16) |  | 37.5 | (18) |  | 0.154 | 0.971 |
| 2DL2 | 24.2 | (100) |  | 17.5 | (10) |  | 25.0 | (12) |  | 0.265 | 0.904 |
| 2DL3 | 99.3 | (410) |  | 100.0 | (57) |  | 100.0 | (48) |  | 1.000 | 1.000 |
| 2DL1 | 99.3 | (410) |  | 100.0 | (57) |  | 100.0 | (48) |  | 1.000 | 1.000 |
| 2DS1 | 35.1 | (145) |  | 29.8 | (17) |  | 37.5 | (18) |  | 0.431 | 0.743 |

P values comparing cases and control are derived from using the Pearson chi-square (χ2) test, continuity correction (when the number of subjects in a cell is <5) or Fisher’ exact test (when the number of subjects in a cell is <1).

**Table S8** The frequency and odds ratio values of KIR-HLA genotypes in healthy control and COVID-19 patients

| KIR+HLA pairs | Healthy control  n = 413 | | Mild cases  n = 57 | | Severe cases  n = 48 | | Mild vs. HC | | Severe vs. HC | |
| --- | --- | --- | --- | --- | --- | --- | --- | --- | --- | --- |
|  | % | (N) | % | (N) | % | (N) | *OR*  (95% *CI*) | *P-* value | *OR*  (95% *CI*) | *P-* value |
| **Inhibitory** |  |  |  |  |  |  |  |  |  |  |
| **2DL1+C2** | 29.3 | (121) | 45.6 | (26) | 33.3 | (16) | 2.256 (1.252-4.068) | **0.013** |  | 0.563 |
| 2DL2+C1 | 23.7 | (98) | 17.5 | (10) | 22.9 | (11) |  | 0.298 |  | 0.900 |
| 2DL3+C1 | 97.3 | (402) | 100.0 | (57) | 95.8 | (46) |  | 0.212 |  | 0.893 |
| **3DL1+Bw4^a^** | 68.8 | (284) | 70.2 | (40) | 52.1 | (25) |  | 0.829 | 0.412 (0.187-0.904) | **0.020** |
| 3DL1+B-80I | 28.1 | (116) | 31.6 | (18) | 29.2 | (14) |  | 0.584 |  | 0.875 |
| 3DL1+B-80T | 31.7 | (131) | 21.1 | (12) | 20.8 | (10) |  | 0.101 |  | 0.121 |
| 3DL1+A-80I | 29.5 | (122) | 38.6 | (22) | 20.8 | (10) |  | 0.164 |  | 0.207 |
| **Activating** |  |  |  |  |  |  |  |  |  |  |
| 2DS1+C2 | 11.1 | (46) | 15.8 | (9) | 10.4 | (5) |  | 0.306 |  | 0.880 |
| 3DS1+Bw4^a^ | 26.2 | (108) | 19.3 | (11) | 18.8 | (9) |  | 0.265 |  | 0.265 |
| 3DS1+B-80I | 9.2 | (38) | 5.3 | (3) | 10.4 | (5) |  | 0.323 |  | 0.990 |
| 3DS1+A-80I | 11.9 | (49) | 8.8 | (5) | 6.3 | (3) |  | 0.493 |  | 0.244 |

P values comparing cases and control are derived from using the Pearson chi-square (χ2) test, continuity correction (when the number of subjects in a cell is <5) or Fisher’ exact test (when the number of subjects in a cell is <1). CI = confidence interval, OR = odds ratio. ORs were computed and 95% CIs were calculated using logistic regression, adjusted for sex, age, hypertension, coronary artery disease and diabetes.

^a^Bw4 motif indicates the presence of one or more HLA-B Bw4-80I, HLA-B Bw4-80T and HLA-A Bw4.
